# OPCML Methylation and the Risk of Ovarian Cancer: A Meta and Bioinformatics Analysis

**DOI:** 10.3389/fcell.2021.570898

**Published:** 2021-03-11

**Authors:** Yang Shao, Jing Kong, Hanzi Xu, Xiaoli Wu, YuePeng Cao, Weijian Li, Jing Han, Dake Li, Kaipeng Xie, Jiangping Wu

**Affiliations:** ^1^Nanjing Maternity and Child Health Care Institute, Nanjing Maternity and Child Health Care Hospital, Women's Hospital of Nanjing Medical University, Nanjing, China; ^2^The First People's Hospital of Zhangjiagang City, The Zhangjiagang Affiliated Hospital of Soochow University, Suzhou, China; ^3^Department of Radiotherapy, The Affiliated Cancer Hospital of Nanjing Medical University, Nanjing, China; ^4^Jiangsu Institute of Cancer Research, The Affiliated Cancer Hospital of Nanjing Medical University, Nanjing, China

**Keywords:** OPCML, methylation, ovarian cancer, risk, progression

## Abstract

**Background:** The association of opioid binding protein cell adhesion molecule-like (OPCML) gene methylation with ovarian cancer risk remains unclear.

**Methods:** We identified eligible studies by searching the PubMed, Web of Science, ScienceDirect, and Wanfang databases. Odds ratios (ORs) and 95% confidence intervals (95% CIs) were used to determine the association of OPCML methylation with ovarian cancer risk. Meta-regression and subgroup analysis were used to assess the sources of heterogeneity. Additionally, we analyzed the Gene Expression Omnibus (GEO) and The Cancer Genome Atlas (TCGA) datasets to validate our findings.

**Results:** Our study included 476 ovarian cancer patients and 385 controls from eight eligible studies. The pooled OR was 33.47 (95% CI = 12.43–90.16) in the cancer group vs. the control group under the random-effects model. The association was still significant in subgroups according to sample type, control type, methods, and sample sizes (all *P* < 0.05). Sensitivity analysis showed that the finding was robust. No publication bias was observed in Begg's (*P* = 0.458) and Egger's tests (*P* = 0.261). We further found that OPCML methylation was related to III/IV (OR = 4.20, 95% CI = 1.59–11.14) and poorly differentiated grade (OR = 4.37; 95% CI = 1.14–16.78). Based on GSE146552 and GSE155760, we validated that three CpG sites (cg16639665, cg23236270, cg15964611) in OPCML promoter region were significantly higher in cancer tissues compared to normal tissues. However, we did not observe the associations of OPCML methylation with clinicopathological parameters and overall survival based on TCGA ovarian cancer data.

**Conclusion:** Our findings support that OPCML methylation is associated with an increased risk of ovarian cancer.

## Introduction

Ovarian cancer is the most frequent cause of death in women with gynecological malignancies. In 2020, 313,959 new ovarian cancer cases were diagnosed and 207,252 new deaths occurred from ovarian cancer in the world (Sung et al., 2021). Most patients are diagnosed at an advanced or incurable stage because of asymptomatic development (Lheureux et al., 2019). Therefore, identifying novel biomarkers for early diagnosis and research on treatment strategies could reduce the incidence of ovarian cancer and improve the survival rate in advanced ovarian cancer patients.

Aberrant gene expression, including the activation of oncogenes and inactivation of tumor suppressor genes (TSGs), plays a vital role in tumorigenesis (Ehrlich, 2002). Emerging studies have shown that DNA methylation is a key mechanism of epigenetic variability in gene expression (Wilson et al., 2007). Compared with normal cells, cancer cells exhibit extensive changes in DNA methylation patterns (Vidal et al., 2017). Cancer cells undergo alterations in 5-methylcytosine distribution including global DNA hypomethylation and hypermethylation of CpG islands (Jones, 2012). Notably, aberrant DNA methylation is not only a well-known mechanism of TSG inactivation but also one of the earliest events in carcinogenesis (Rauscher et al., 2015; Widschwendter et al., 2017). DNA methylation is considered a promising biomarker for cancer diagnosis and the prediction of treatment and prognosis (Szyf, 2012; Widschwendter et al., 2017). Intriguingly, the Food and Drug Administration (FDA) has approved the first methylation-based assay in colorectal cancer screening and early detection (Song et al., 2017; Widschwendter et al., 2018). Therefore, evaluating the alteration of DNA methylation in ovarian cancer is urgent.

Opioid binding protein cell adhesion molecule-like (OPCML) gene at 11q25 is a glycosyl-phosphatidylinositol (GPI)-anchored cell adhesion-like molecule and belongs to the IgLON family (Wu and Sood, 2012). OPCML demonstrates tumor-suppressor function in epithelial ovarian cancer both *in vitro* and *in vivo* (Sellar et al., 2003). Subsequently, the same team described the mechanism underlying the phenotype of OPCML. It negatively regulates a specific receptor tyrosine kinase (RTK) repertoire comprising erythropoietin-producing hepatocellular receptor-2 (EPHA2), fibroblast growth factor receptor-1 (FGFR1), fibroblast growth factor receptor-3 (FGFR3), human epidermal growth factor receptor-2 (HER2), and human epidermal growth factor receptor-4 (HER4) receptors by binding to the extracellular domains of RTKs, thus promoting their degradation *via* a polyubiquitination-associated proteasomal mechanism and leading to growth inhibition (McKie et al., 2012). Additionally, they revealed that a recombinant protein based on OPCML is an active anticancer agent preclinical *in vivo*. Another study showed that OPCML restoration interfered with HER2-EGFR heterodimer formation and potentiated lapatinib and erlotinib therapy in ovarian and breast cancer cell lines overexpressing HER2 (Zanini et al., 2017). Recent researches on OPCML protein reveal that OPCML is a potential anti-cancer therapy (Birtley et al., 2019; Simovic et al., 2019). Considering the biological and clinical effects of OPCML, research on the mechanism of aberrant expression patterns may benefit ovarian cancer patients from OPCML-based therapy.

Many studies have indicated that OPCML DNA hypermethylation frequently occurs in ovarian cancers. However, the findings remain unclear regarding the small population and different methods in previous studies (Czekierdowski et al., 2006; Zhang et al., 2006, 2014; Chen et al., 2007; Liu et al., 2008; Zhou et al., 2011, 2014a,b; Wang et al., 2015, 2017; Xing et al., 2015). Therefore, we performed a meta-analysis to identify the association of OPCML methylation with ovarian cancer risk. We further assessed whether OPCML methylation was associated with other clinicopathological parameters in eligible studies, such as age, stage, histological type, and tumor differentiation. Additionally, we validated our results based on the Gene Expression Omnibus (GEO) and The Cancer Genome Atlas (TCGA) ovarian cancer datasets.

## Materials and Methods

### Literature Search Strategy

A systematic literature search was performed in the PubMed, Web of Science, ScienceDirect, and Wanfang databases using the following keywords and search items: (ovarian OR ovary) AND (cancer OR carcinoma OR tumor) AND (OPCML methylation). The search was updated until November 17, 2020.

### Study Selection

The inclusion criteria were as follows: (1) studies primarily evaluating the association between OPCML methylation and ovarian cancer, (2) studies including case and control populations, (3) the incidence of OPCML methylation in case and control groups, and (4) studies using human tissues or blood. Based on the inclusion criteria, the titles and abstracts from the preliminary search were evaluated. Subsequently, all related studies were evaluated as full-text papers. Exclusion criteria were (1) book section, conference abstracts, and reviews, (2) studies with insufficient data to provide the methylation frequencies in cancer and control groups, and (3) studies without human samples. In the case of duplicated publications from the shared cohort, we selected the most complete information to be included in the meta-analysis.

### Data Extraction and Quality Assessment

The following information was extracted for each eligible study: the first author's name, publication year, country, age, number of methylated and unmethylated samples in cancer and control groups, sample type, control type, methylation detection methods, and other clinicopathological parameters. In our study, controls included adjacent non-cancerous ovarian tissues (AT), tissue and blood samples from benign ovarian tumor (BOT) and other cancer-free patients (NT). Additionally, we used the Newcastle-Ottawa Scale (NOS) to assess the quality. The NOS includes the selection of the research groups (four stars), comparison of the groups (two stars), and ascertainment of the outcome (three stars).

### Bioinformatics Analysis

Two DNA methylation data were downloaded from the Gene Expression Omnibus (GEO) database: GSE146552 (20 high grade serous ovarian cancers, 16 epithelial layer by mechanical scraping of resected fimbriae fallopian tubes and ovaries) and GSE155760 (23 high-grade serous ovarian carcinoma, 11 fallopian tube mucosa from cancer-free normal control). The GEO2R online analysis tool (https://www.ncbi.nlm.nih.gov/geo/geo2r/) was used to identify the differential DNA methylated sites (*P* < 0.05) between cancer tissues and normal tissues. For the cancer genome atlas (TCGA) ovarian serous cystadenocarcinoma (OV) data, we downloaded the OPCML expression data (log2-transformed RSEM normalized count) based on RNA sequencing (RNA-Seq), OPCML-associated DNA methylation data (cg03923934, cg25853078) based on HumanMethylation27 and the clinical data including age, stage, grade, and overall survival from the website (https://xena.ucsc.edu/).

### Statistical Analysis

We calculated the pooled odds ratios (ORs) and 95% confidence intervals (95% CIs) to evaluate the association between OPCML methylation and ovarian cancer risk. The heterogeneity of studies was evaluated by the χ^2^-based *Q*-test. When the *P* > 0.05, the fixed-effects model was used to combine the effect size; otherwise, the random-effects model was adopted. We conducted meta-regression to explore sources of heterogeneity and further performed a subgroup analysis to evaluate the source of the heterogeneity. The contribution of each study to our findings was examined according to sensitivity analysis. We used the Begg's rank correlation method (Begg and Mazumdar, 1994) and a funnel plot for Egger's test (Egger et al., 1997) to evaluate publication bias.

The correlation between OPCML methylation and its expression in TCGA was calculated by Spearman's rank correlation. Kruskal-Wallis and Wilcoxon-Mann-Whitney *U* tests were applied for comparisons, as appropriate. Survival analysis was performed using the log-rank test and a Cox regression model. Hazard ratios (HRs) and corresponding 95% CIs were calculated to assess the associations of factors with overall survival. All statistical tests were two-sided and performed using the R software (version 3.6). *P-*value < 0.05 were considered statistically significant.

## Results

### Study Characteristics

**Figure 1:**
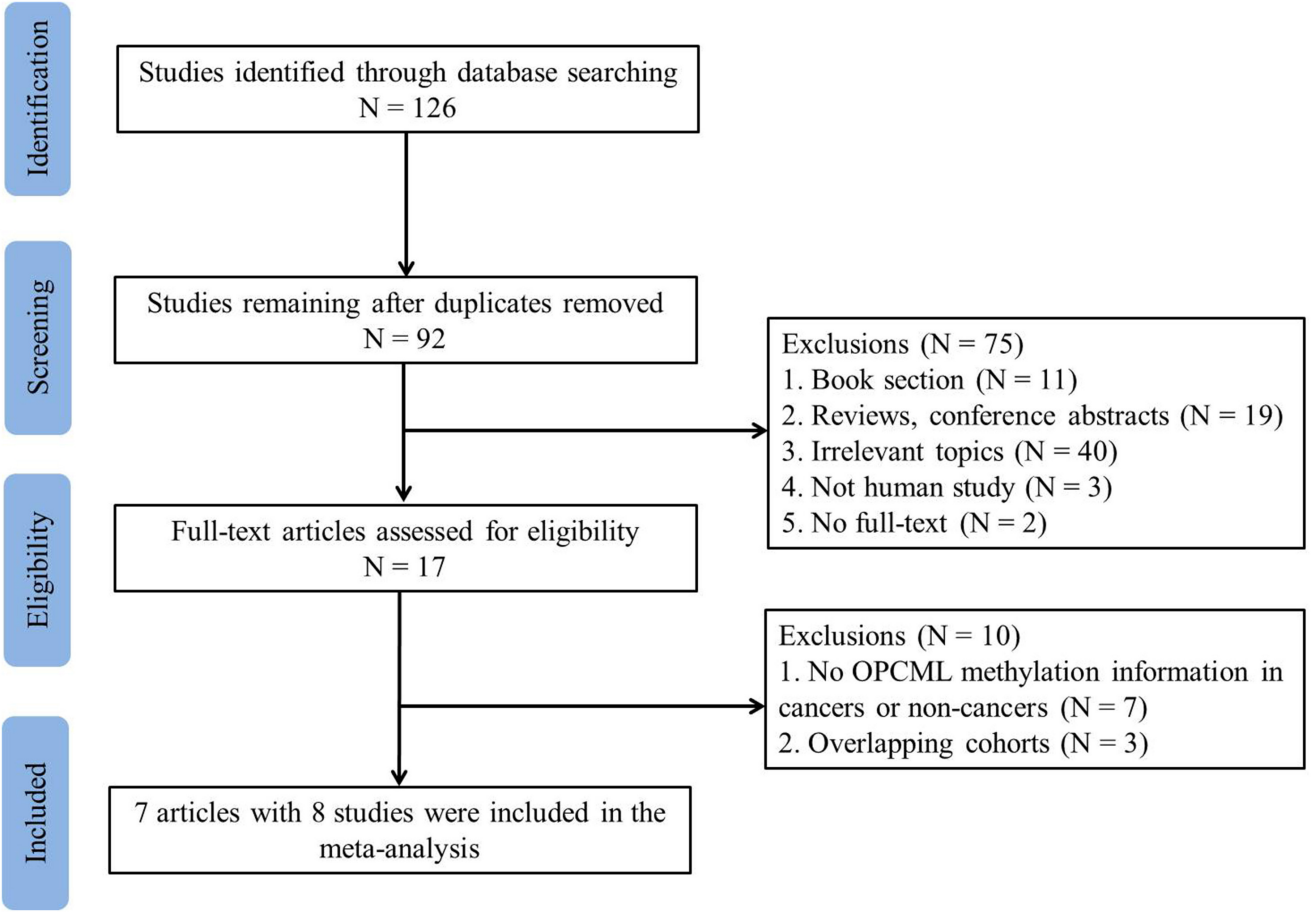
Flowchart of study selection in the meta-analysis.

The detailed information of how we selected relevant articles is shown in Figure 1. We initially identified 126 articles from PubMed, Web of Science, ScienceDirect, and Wanfang databases and excluded 34 duplicated articles. After reviewing 92 articles based on titles and abstracts, 75 articles did not meet the selection criteria, leaving 17 studies for detailed full-text evaluation. Seven articles were excluded because they did not report the methylation frequencies in cancers or controls. Three articles were excluded because of overlapping cohorts (Zhou et al., 2011; Zhang et al., 2014; Wang et al., 2017). The study performed by Zhou et al. (2014a) included two sample types, tissue and serum. Thus, we considered this paper as two separate studies. Therefore, seven eligible articles with eight studies including 476 ovarian cancer patients and 385 controls were included in the meta-analysis.

The characteristics of the eight studies are summarized in Table 1. Seven studies were performed in China and one study was performed in Poland. The cases comprised of cancer tissues and blood from ovarian cancer patients. Among the eight included studies, six studies clearly described that the cases were primary tumor patients whereas two studies (Wang et al., 2015; Xing et al., 2015) did not. In the study performed by Liu et al. (2008), there are 41 metastatic tissues of pelvic and abdomen cavity. We provided this in Table 1. However, we did not include these samples when we calculated the pooled OR. The controls in these studies were not matched to cases for any features. OPCML promoter methylation was detected in all eligible articles. There are four studies that used methylation-specific polymerase chain reaction (MSP), three used restriction enzyme-related analysis [methylation-sensitive restriction enzyme-polymerase chain reaction (MSRE-PCR) and restriction enzyme cut analysis], and one study used cationic conjugated polymer (CCP)-based fluorescence resonance energy transfer (FRET) to detect OPCML methylation in ovarian cancer and controls. According to the NOS criteria, the quality of these studies was six, indicating a relatively high quality. Detailed features are shown in Supplementary Table 1.

**Table 1:** Characteristics of included studies.

**References**	**Country**	**Age (mean or median)**	**Cancer group**		**Control group**		**Sample type**	**Control type**	**Method**	**Methylation frequency of OPCML associated with other clinicopathological parameters**
			**M**	**U**	**M**	**U**				
Wang et al. (2015)	China	Cancer, 54.06 years. Benign, 33.95 years. Healthy, 46.17 years.	64	7	11^a^	112^a^	Serum	BOT&NT	MSP	FIGO stage: I/II (33/39), III/IV (30/32).
Xing et al. (2015)	China	NR	27	8	2	9	Tissues	NT	CCP-based FRET	Age: <60 years (23/28), ≥60 years (4/7). FIGO stage: I/II (5/11), III/IV (22/24). Histological type: serous (22/26), others (5/9)^b^. Grade of differentiation^c^: High-grade serous (19/21), others (8/14) Ascites: positive (21/24), Negative (6/11). CA125: >200 U/mL (21/25), <200 U/mL (6/10)
Zhou et al. (2014a)	China	Cancer and normal, 50 years.	39	6	0^d^	40^d^	Tissues	NT	MSRE-PCR	Tissues FIGO stage: I/II (5/10), III/IV (34/35). Histological type: serous (18/20), others (21/25)^e^. Tumor differentiation: Well and moderately differentiated (10/16), Poorly differentiated (29/29).
		Healthy, 50 years.	36	9	0^d^	20^d^	Serum	NT	MSRE-PCR	Serum FIGO stage: I/II (5/10), III/IV (31/35). Histological type: serous (17/20), others (19/25)^e^. Tumor differentiation: Well and moderately differentiated (9/16), Poorly differentiated (27/29).
Zhou et al. (2014b)	China	Cancer, 54 years. Benign, 53 years. Normal, 54 years.	80	22	0^f^	30^f^	Tissues	NT	MSP	Age: <55 years (39/54), ≥55 years (41/48). Pathological stage^*^: I/II (31/44), III/IV (49/58). Histological type^b^: serous (42/57), others (38/45).
			–	–	28^f^	57^f^	Tissues	BOT	MSP	
Czekierdowski et al. (2006)	Poland	NR^g^	20	23	0	4	Tissues	NT	MSP	FIGO stage: I/II (4/5), III/IV (16/38). Histological type: serous (8/15), others (12/28)^h^. Histological Grading: G1+G2 (11/20), G3 (9/23)^k^
Zhang et al. (2006)	China	Cancer, 51.5 years. Benign, 39.0 years. Normal, 46.5 years.	32	40	0	20	Tissues	NT	Restriction enzyme cut	
					0	17	Tissues	BOT	Restriction enzyme cut	
Liu et al. (2008)^i^	China	Cancer, 53.8 years. Normal, 53.2 years.	30	33	0	20	Tissues	NT	MSP	FIGO stage: I/II (2/22), III/IV (16/41). Histological type: serous (20/34), others (10/29)^j^. Grading: Well and Moderately differentiated (6/36), Poorly differentiated (12/27)
					2	13	Tissues	AT		

*M, methylation; U, unmethylation; BOT, benign ovarian tissues or blood; NT, normal ovarian tissues or blood of cancer-free patients or healthy people; AT, Adjacent non-cancerous ovarian tissues; MSP, methylation-specific polymerase chain reaction; MSRE-PCR, methylation-sensitive restriction enzyme-polymerase chain reaction; CCP-based FRET, cationic conjugated polymer (CCP)-based fluorescence resonance energy transfer (FRET); FIGO, International Federation of Gynecology and Obstetrics. NR, not reported the median or mean age in all patients*.

a *The control group include 43 benign ovarian carcinoma and 80 healthy women in this study*.

b *Others included Mucinous, Clear cell, and Endometrioid carcinomas*.

c *In further analysis, we regarded the high-grade serous as Poorly differentiated., others as well and moderately differentiated*.

d *This study include 45 ovarian tumor tissues, 40 normal ovarian tissues, and 20 serum samples*.

e *Others included Mucinous, Clear cell, Endometrioid, and undifferentiated carcinomas*.

f *This study include 102 ovarian cancer tissues, 85 benign ovarian tumor, and 30 normal ovarian*.

* *Pathological stage was classified according to the tumor, lymph node, and metastasis (TNM) classification of the American Joint Committee on Cancer (AJCC)*.

g *This study only reported the mean ages of different histological type, but not reported the mean age of cancer in tumor and normal tissue*.

h *Others included Mucinous, Clear cell, Endometrioid, undifferentiated, metastatic, and other carcinomas*.

i *This study include 63 epithelial ovarian cancer tissues, 41 Metastatic tissues of pelvic and abdomen cavity, 15 Adjacent non-cancerous ovarian tissues, 20 Normal ovairan tissues*.

j *Others included Mucinous, Clear cell, and Endometrioid carcinomas*.

k *In further analysis, we regarded the G1+G2 as well and moderately differentiated, G3 as poorly differentiated*.

### Relationship Between OPCML Methylation and Ovarian Cancer Risk

**Figure 2:**
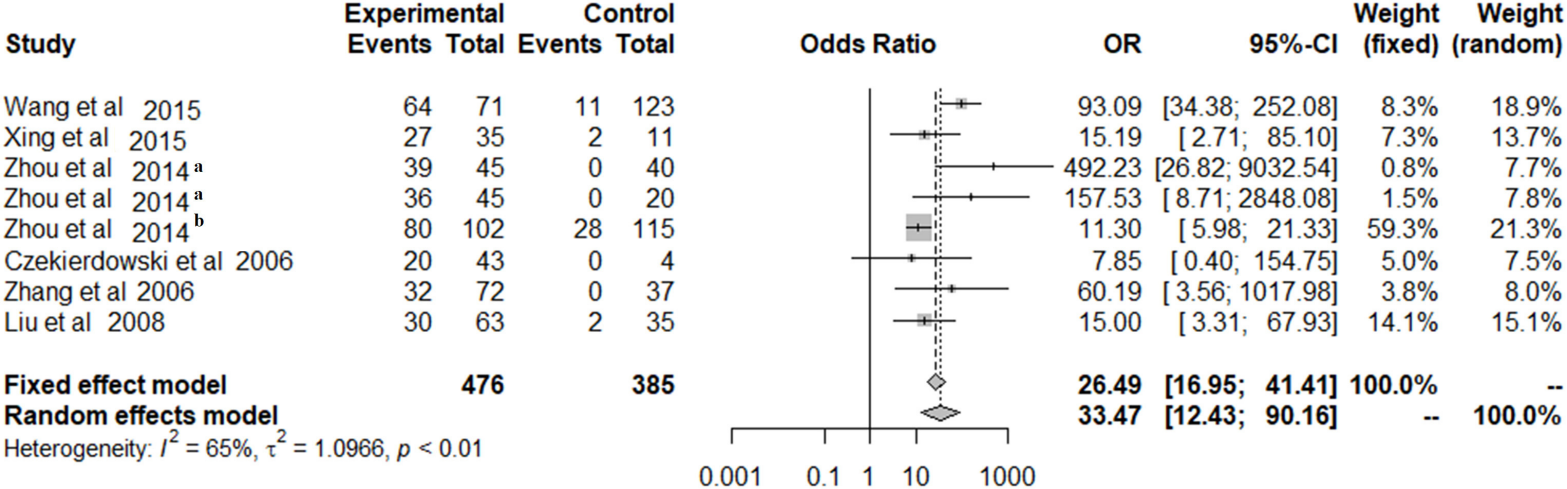
The summary estimates for OPCML methylation frequency associated with ovarian cancer in the meta-analysis.

Because of significant heterogeneity among the included studies, a random-effects model was used to evaluate the effect size (*P* < 0.01; *I*^2^ = 65%; Figure 2). In the overall meta-analysis, the OPCML methylation frequency was significantly associated with an increased risk of ovarian cancer (summary OR: 33.47; 95% CI = 12.43–90.16; *P* < 0.001; Figure 2).

### Meta-Regression and Subgroup Analysis

To explore the potential sources of heterogeneity, we performed multiple regression model using six variables (publication year, country, sample type, control type, methods, and sample size). However, the source of heterogeneity was not observed among these factors (all *P* > 0.05; Table 2). We also conducted subgroup analysis to assess the source of the heterogeneity according to country, sample type, sample size, control type, and method (Table 3). In the subgroup analysis of country, the OR was 38.17 (95% CI = 13.21–110.28) in China under the random-effects model. The OR was 102.86 (95% CI = 39.37–268.74) under the fixed-effects model in the serum subgroup, and 18.22 (95% CI = 10.93–30.36) under the fixed-effects model in the tissues subgroup. In the subgroup analysis based on the control type, the OR was 52.77 (95% CI = 16.92–164.55) in the NT group under the fixed-effects model and 35.50 (95% CI = 6.83–197.47) in the NT and BOT groups under the random-effects model. In the subgroup analysis of methods, the OR was 21.78 (95% CI = 6.10–77.79) in the MSP group under the random-effects model and 139.45 (95% CI = 24.06–808.26) in the restriction enzyme-related analysis group under the fixed-effects model. The OR for the sample size subgroup was 34.19 (95% CI = 14.11–82.86) in the <100 subgroup under the fixed-effects model and 35.50 (95% CI = 6.38–197.47) in the ≥100 subgroup under the random-effects model.

**Table 2:** Meta-regression analysis.

**Heterogeneity sources**	**Coefficient**	**95% CI**	***P***
Publication year	0.814	(−0.751, 2.380)	0.308
Country	10.538	(−9.721, 30.795)	0.308
Sample type	−0.716	(−3.370, 1.939)	0.597
Control type	4.851	(−4.042,13.744)	0.285
Method	3.748	(−1.419, 8.916)	0.155
Sample size	−0.001	(−0.020, 0.018)	0.889

**Table 3:** Subgroup analysis of associations between *OPCML* methylation and ovarian cancer risk.

**Group**	**Studies**	**Case**		**Control**		**Fixed effects model**	**Random effects model**	**Heterogeneity**	
	***n***	**M**	**U**	**M**	**U**	**OR (95% CI)**	**OR (95% CI)**	***I*^**2**^ (%)**	***P***
Country									
China	7	308	125	43	338	27.47 (17.52, 43.08)	38.17 (13.21, 110.28)	70	<0.01
Poland	1	20	23	0	4	7.85 (0.40, 154.75)	7.85 (0.40, 154.75)	–	–
Sample type									
Serum	2	100	16	11	132	102.86 (39.37, 268.74)	98.42 (38.37, 252.45)	0	0.73
Tissues	6	228	132	32	210	18.22 (10.93, 30.36)	19.13 (7.71, 47.44)	39	0.15
Control type									
NT	4	122	46	2	73	52.77 (16.92, 164.55)	47.94 (7.38, 311.27)	53	0.09
NT&BOT	3	176	69	39	236	23.41 (13.96, 39.25)	35.50 (6.38, 197.47)	84	<0.01
AT&NT	1	30	33	2	33	15.00 (3.31, 67.93)	15.00 (3.31, 67.93)	–	–
Methods									
MSP	4	194	85	41	236	19.52 (11.94, 31.92)	21.78 (6.10, 77.79)	76	<0.01
CCP-based FRET	1	27	8	2	9	15.19 (2.71, 85.10)	15.19 (2.71, 85.10)	–	–
Restriction enzyme related methods	3	107	55	0	97	139.45 (24.06, 808.26)	163.86 (31.13, 862.61)	0	0.59
Sample size									
<100	5	152	79	4	106	34.19 (14.11, 82.86)	32.63 (8.42, 126.53)	45	0.12
≥100	3	176	69	39	236	23.41 (13.96, 39.25)	35.50 (6.38, 197.47)	84	<0.01

*M, methylation; U, unmethylation; BOT, benign ovarian tissues; NT, normal ovarian tissues of cancer-free patients or healthy people; AT, Adjacent non-cancerous ovarian tissues; MSP, methylation-specific polymerase chain reaction; CCP-based FRET, a cationic conjugated polymer (CCP)-based fluorescence resonance energy transfer (FRET); Restriction enzyme related methods include methylation-sensitive restriction enzyme-polymerase chain reaction and restriction enzyme cut analysis*.

### Sensitivity Analysis

**Figure 3:**
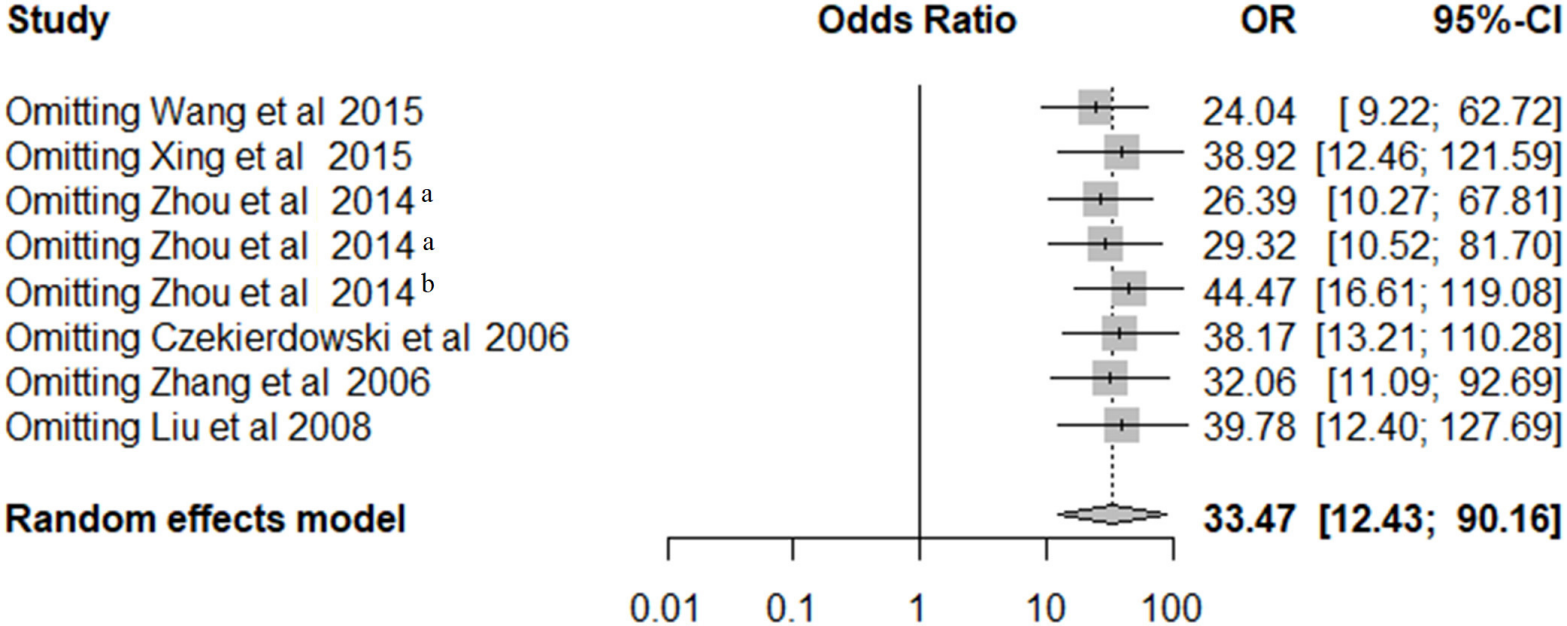
The sensitivity analysis of pooled OR for OPCML methylation and ovarian cancer risk under the random-effects model.

As shown in Figure 3, leave-one-out sensitivity analysis was performed by removing a single study at a time under the random-effects model to assess the stability of the results. Our results showed that the ORs ranged from 24.04 (95% CI = 9.22–62.72) to 44.47 (95% CI = 16.61–119.08).

### Publication Bias

**Figure 4:**
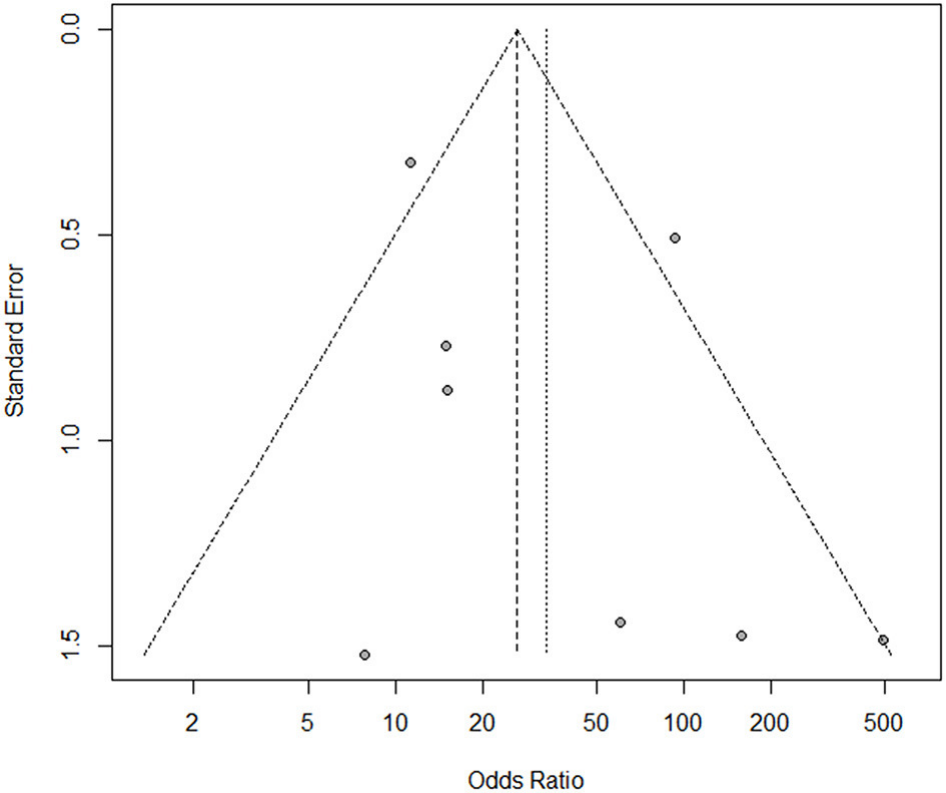
The funnel plot for assessment of publication bias in the meta-analysis.

Begg's and Egger's tests were performed to estimate the publication bias of the included studies. The funnel plot was approximately symmetric (Figure 4), indicating no publication bias in Begg's (*P* = 0.458) or Egger's tests (*P* = 0.261).

### Relationship Between OPCML Methylation and Clinicopathological Features

**Figure 5:**
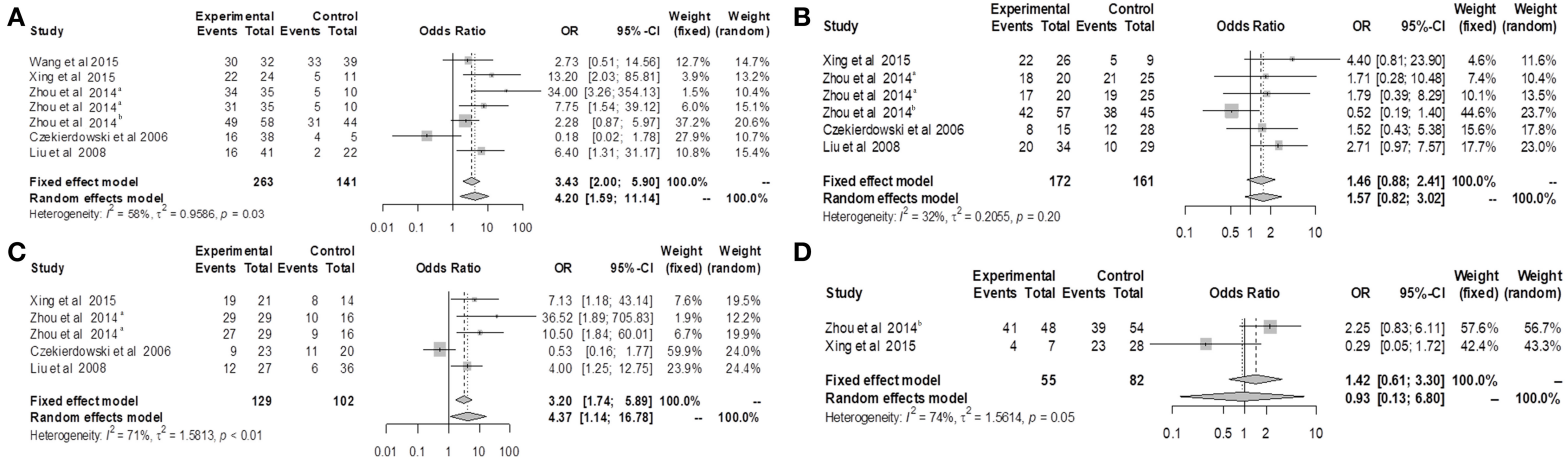
Forest plots for OPCML methylation and clinicopathological features of ovarian cancer patients. **(A)** Forest plot for the relationship between OPCML methylation and III/IV stage. **(B)** Forest plot for the relationship between OPCML methylation and serous ovarian cancer. **(C)** Forest plot for the relationship between OPCML methylation and poorly differentiated tumor. **(D)** Forest plot for the relationship between OPCML methylation and older age.

We divided the ovarian cancer patients into groups based on clinical features including stage, histological type, tumor differentiation, and age, and then further valuated the associations between other variables and OPCML methylation (Figure 5). The findings suggested that methylation was related to III/IV (OR = 4.20; 95% CI = 1.59–11.14) and poorly differentiated tumor (OR = 4.37; 95% CI = 1.14–16.78), while methylation was not related to serous histology (OR = 1.46; 95% CI = 0.88–2.41) or older age (OR = 1.42; 95% CI = 0.61–3.30).

### Validation of the Results in Multiple Public Databases

**Figure 6:**
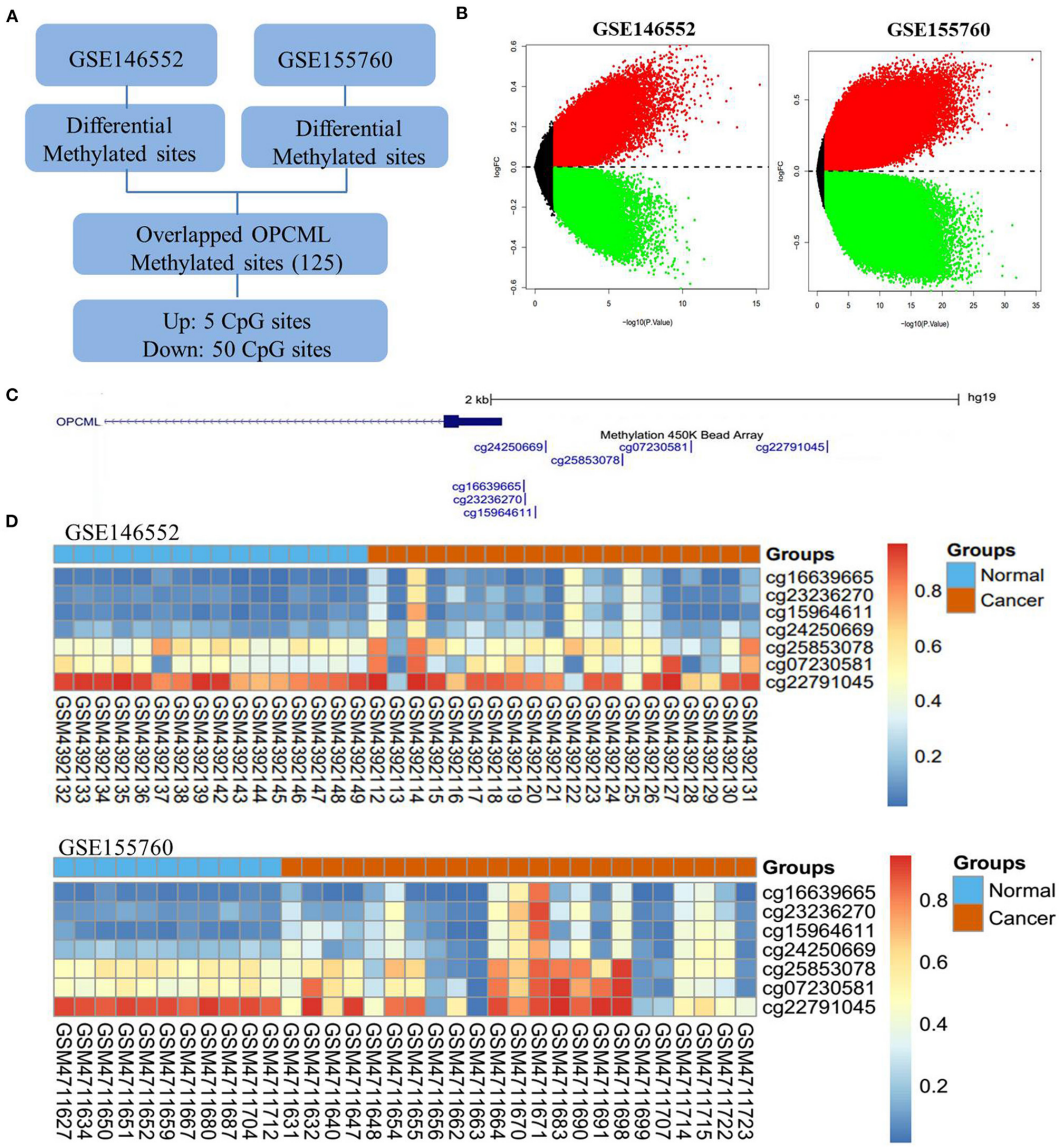
The OPCML methylation status in ovarian cancer tissues and normal tissues. **(A)** The workflow of differential methylated sites in GSE146552 and GSE155760. **(B)** Volcano plots of differential methylated sites based on GSE146552 and GSE155760. **(C)** There are seven CpG sites in OPCML promoter regions. **(D)** The methylation status of seven CpG sites in GSE146552 and GSE155760. Among them, three differential methylated sites (cg16639665, cg23236270, cg15964611) were observed in both GSE146552 and GSE155760.

**Figure 7:**
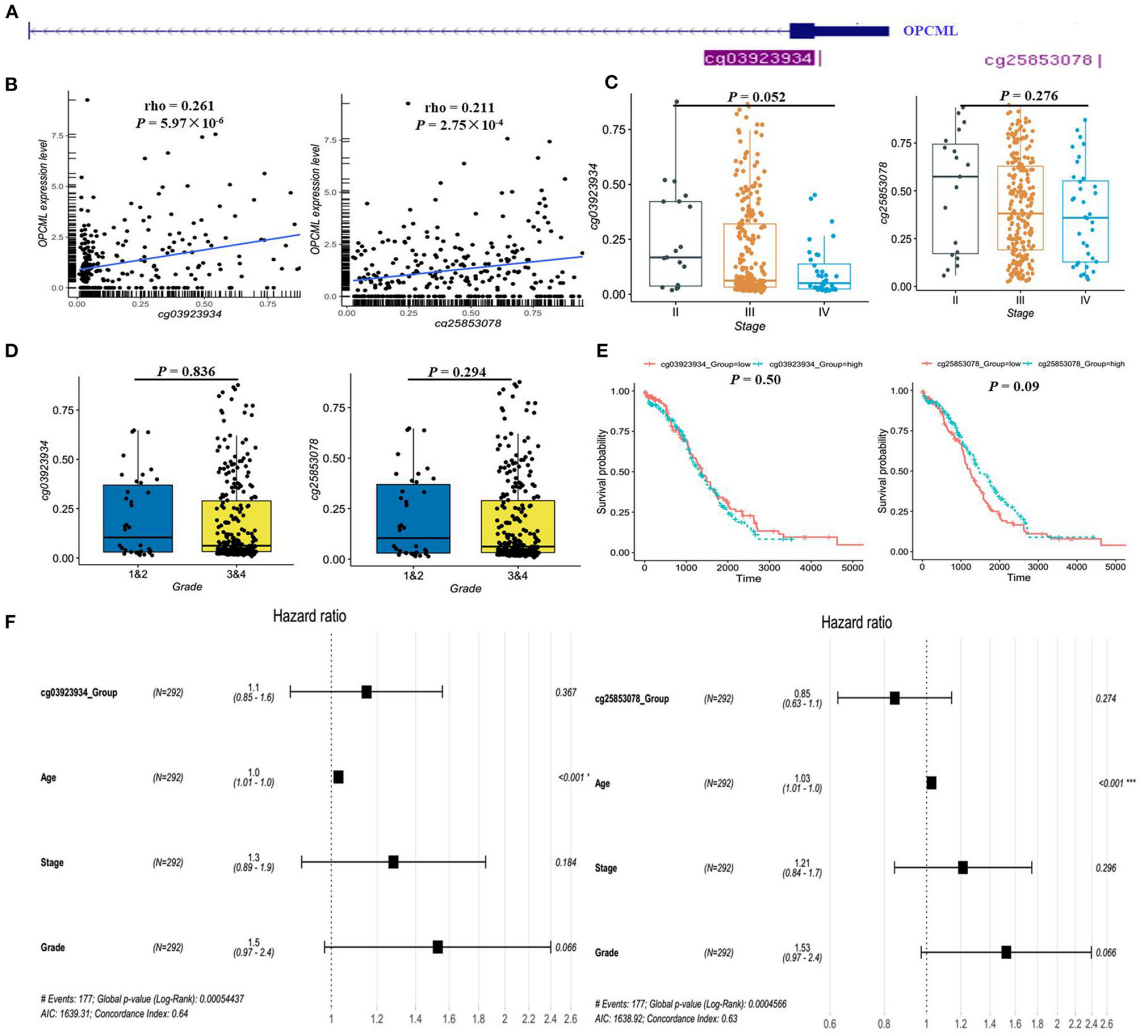
The methylation status of OPCML-associated CpG sites in the TCGA dataset. **(A)** There are only two CpG sites from HumanMethylation27k, and the location of two CpG sites in OPCML. **(B)** There are significantly positive associations between two CpG sites and OPCML expression. **(C)** The methylation levels were not significantly associated with clinical stage. **(D)** The methylation levels were not significantly associated with clinical grade. **(E)** No significant associations were observed in patient with different methylation levels using log-rank analysis. **(F)** No significant associations were observed in patient with different methylation levels using cox analysis.

Based on GSE146552 and GSE155760 datasets, we evaluated the DNA methylation profiling in ovarian cancer tissues and normal tissues (Figure 6A). We firstly analyzed the differential methylated sites between cancer tissues and normal tissues (Figure 6B). Then, we found that methylation levels of 55 differential methylated CpG sites were consistent in two datasets (Table 4). Among them, three CpG sites (cg16639665, cg23236270, cg15964611) were located in OPCML promoter regions (Figures 6C,D, all *P* < 0.05). In consistent with the results of meta-analysis, the methylation levels of three CpG sites were significantly higher in cancer tissues compared to normal tissues. Based on the TCGA ovarian cancer datasets, two OPCML-associated CpG sites (cg03923934, cg25853078, Figures 7A,B) were significantly associated with OPCML expression (rho = 0.261, *P* = 5.97 × 10^−6^; rho = 0.211, *P* = 2.75 × 10^−4^). However, we did not observe the significant associations for the clinicopathological parameters and overall survival time (Figures 7C–F).

**Table 4:** Consistently differential DNA methylated sites (*P* < 0.05 and the same direction of logFC in both datasets) between ovarian cancer tissues and normal tissues based on GEO DNA methylation datasets.

**ID**	**chrom**	**chromStart**	**chromEnd**	**GSE155760**		**GSE146552**		**Gene**
				**logFC**	***P-*value**	**logFC**	***P-*value**	
cg01875106	chr11	132287341	132287343	−0.6360	1.050 × 10^−12^	−0.2740	1.500 × 10^−4^	OPCML
cg11500790	chr11	132321480	132321482	−0.5980	1.400 × 10^−7^	−0.3440	8.150 × 10^−5^	OPCML
cg05806819	chr11	132432636	132432638	−0.0195	3.980 × 10^−2^	−0.0523	3.340 × 10^−2^	OPCML
cg03784083	chr11	132513894	132513896	−0.4300	4.610 × 10^−4^	−0.2160	4.350 × 10^−3^	OPCML
cg08080489	chr11	132526946	132526948	−0.5720	6.510 × 10^−8^	−0.2800	1.240 × 10^−4^	OPCML
cg17242596	chr11	132527295	132527297	−0.6000	3.930 × 10^−8^	−0.3990	1.650 × 10^−5^	OPCML
cg06967998	chr11	132527437	132527439	−0.6200	1.490 × 10^−7^	−0.3880	2.850 × 10^−5^	OPCML
cg10376306	chr11	132588617	132588619	−0.3060	7.050 × 10^−4^	−0.1590	5.340 × 10^−3^	OPCML
cg12156287	chr11	132662865	132662867	−0.4030	3.800 × 10^−12^	−0.1600	2.330 × 10^−2^	OPCML
cg18944144	chr11	132673498	132673500	−0.5470	1.730 × 10^−6^	−0.2650	1.120 × 10^−3^	OPCML
cg05035315	chr11	132691483	132691485	−0.6190	2.010 × 10^−9^	−0.3730	7.990 × 10^−6^	OPCML
cg13136241	chr11	132709827	132709829	−0.3190	1.080 × 10^−3^	−0.1770	1.210 × 10^−2^	OPCML
cg26824847	chr11	132728087	132728089	−0.5170	2.470 × 10^−10^	−0.3810	1.270 × 10^−6^	OPCML
cg19743254	chr11	132735813	132735815	−0.3410	1.090 × 10^−3^	−0.1950	1.950 × 10^−2^	OPCML
cg10966440	chr11	132745700	132745702	−0.6570	7.910 × 10^−11^	−0.4670	8.810 × 10^−7^	OPCML
cg23813681	chr11	132772322	132772324	−0.4060	1.880 × 10^−4^	−0.1810	2.100 × 10^−2^	OPCML
cg16813466	chr11	132811377	132811379	−0.4090	6.750 × 10^−5^	−0.2440	1.190 × 10^−3^	OPCML
cg07649562	chr11	132813503	132813505	0.0662	4.840 × 10^−2^	0.0419	2.210 × 10^−2^	OPCML
cg18710784	chr11	132813562	132813564	0.2530	9.680 × 10^−3^	0.2100	1.020 × 10^−3^	OPCML
cg15618210	chr11	132816802	132816804	−0.6010	9.760 × 10^−8^	−0.3020	5.000 × 10^−4^	OPCML
cg02993944	chr11	132831380	132831382	−0.3230	1.030 × 10^−3^	−0.1520	1.250 × 10^−2^	OPCML
cg16123848	chr11	132853100	132853102	−0.4960	9.470 × 10^−6^	−0.3320	3.560 × 10^−4^	OPCML
cg11747462	chr11	132869599	132869601	−0.2650	6.650 × 10^−4^	−0.1320	2.100 × 10^−2^	OPCML
cg03887438	chr11	132910641	132910643	−0.3410	3.090 × 10^−13^	−0.1540	5.610 × 10^−3^	OPCML
cg18413062	chr11	132931731	132931733	−0.4250	1.660 × 10^−9^	−0.2350	5.720 × 10^−4^	OPCML
cg13296579	chr11	132934961	132934963	−0.1630	3.160 × 10^−2^	−0.0885	3.160 × 10^−2^	OPCML, AP004782.1
cg20417723	chr11	132935119	132935121	−0.2570	4.250 × 10^−3^	−0.1440	1.560 × 10^−2^	OPCML, AP004782.1
cg26906285	chr11	132935462	132935464	−0.1720	1.820 × 10^−2^	−0.1560	3.880 × 10^−3^	OPCML, AP004782.1
cg05886537	chr11	132936427	132936429	−0.3250	2.030 × 10^−3^	−0.1770	3.810 × 10^−3^	OPCML, AP004782.1
cg08945802	chr11	132946454	132946456	−0.1980	1.710 × 10^−2^	−0.0864	4.530 × 10^−2^	OPCML
cg05807849	chr11	132946691	132946693	−0.4100	2.210 × 10^−5^	−0.2090	7.820 × 10^−3^	OPCML
cg11983942	chr11	132946933	132946935	−0.3910	8.510 × 10^−8^	−0.1930	8.810 × 10^−4^	OPCML
cg08357627	chr11	132949125	132949127	−0.5150	3.900 × 10^−11^	−0.2480	3.460 × 10^−4^	OPCML
cg11515095	chr11	132949436	132949438	−0.3320	1.500 × 10^−3^	−0.1840	8.340 × 10^−3^	OPCML
cg10335834	chr11	132949780	132949782	−0.2800	1.370 × 10^−3^	−0.1570	1.030 × 10^−2^	OPCML
cg15702349	chr11	132950103	132950105	−0.1970	4.550 × 10^−3^	−0.0989	1.620 × 10^−2^	OPCML
cg26237595	chr11	132955641	132955643	−0.2940	6.960 × 10^−3^	−0.1910	6.130 × 10^−3^	OPCML
cg01311955	chr11	132965249	132965251	−0.2780	1.140 × 10^−4^	−0.2670	1.270 × 10^−3^	OPCML
cg20972294	chr11	133005918	133005920	−0.3710	9.260 × 10^−4^	−0.1940	1.420 × 10^−2^	OPCML
cg12697833	chr11	133085432	133085434	−0.5370	9.690 × 10^−8^	−0.2850	1.670 × 10^−3^	OPCML
cg26311804	chr11	133155302	133155304	−0.3740	4.070 × 10^−4^	−0.1680	6.610 × 10^−3^	OPCML
cg04913291	chr11	133187955	133187957	−0.4150	9.420 × 10^−6^	−0.2830	1.090 × 10^−3^	OPCML
cg22209355	chr11	133208306	133208308	−0.3650	6.770 × 10^−4^	−0.2050	6.410 × 10^−4^	OPCML
cg05131064	chr11	133228365	133228367	−0.2820	2.300 × 10^−3^	−0.1370	2.400 × 10^−2^	OPCML
cg08872013	chr11	133229156	133229158	−0.2390	3.790 × 10^−3^	−0.1100	2.460 × 10^−2^	OPCML
cg20886183	chr11	133230390	133230392	−0.2530	4.390 × 10^**−3**^	−0.1980	1.230 × 10^−2^	OPCML, OPCML-IT1
cg01880176	chr11	133230949	133230951	−0.3070	7.980 × 10^**−5**^	−0.1540	4.180 × 10^−3^	OPCML, OPCML-IT1
cg06379368	chr11	133231146	133231148	−0.2650	1.750 × 10^**−3**^	−0.1630	1.330 × 10^−2^	OPCML, OPCML-IT1
cg13974487	chr11	133231880	133231882	−0.2720	1.790 × 10^**−3**^	−0.1710	1.340 × 10^−3^	OPCML, OPCML-IT1
cg18204273	chr11	133232926	133232928	−0.1850	2.260 × 10^**−2**^	−0.1540	1.490 × 10^−2^	OPCML, OPCML-IT1
cg01140660	chr11	133233199	133233201	−0.4660	1.190 × 10^**−4**^	−0.2160	5.430 × 10^−3^	OPCML, OPCML-IT1
cg09531376	chr11	133234072	133234074	−0.3370	1.850 × 10^**−3**^	−0.1560	4.630 × 10^−3^	OPCML, OPCML-IT1
**cg16639665**	chr11	133402496	133402498	0.1380	3.450 × 10^−2^	0.1130	1.090 × 10^−2^	OPCML
**cg23236270**	chr11	133402499	133402501	0.1630	2.090 × 10^−2^	0.1150	4.540 × 10^−3^	OPCML
**cg15964611**	chr11	133402544	133402546	0.2070	2.630 × 10^−3^	0.1080	3.180 × 10^−2^	OPCML

*Bold values represent CpG sites in OPCML promoter region*.

## Discussion

In the present study, we pooled eight studies together and compared the frequency of OPCML methylation in 476 ovarian cancers with 385 non-malignant tissues or blood samples. The results indicated that OPCML methylation was associated with ovarian cancer risk. The association was still significant in subgroups according to sample type, control type, methods, and sample size. Additionally, we observed significant associations between OPCML methylation and clinical stages and the grade of differentiation in ovarian cancer based on meta-analysis.

OPCML widely downregulated in many tumor types including brain tumors (Reed et al., 2007), non-small cell lung carcinoma (Tsou et al., 2007), bladder cancer (Duarte-Pereira et al., 2011), cholangiocarcinoma (Sriraksa et al., 2011), nasopharyngeal, esophageal, gastric, hepatocellular, colorectal, breast, cervical cancers, and ovarian cancer (Cui et al., 2008). Zanini et al. (2017) found that OPCML expression is associated with better progression free survival in HER2-positive ovarian cancer patients. Notably, exogenous recombinant OPCML protein inhibited ovarian cancer cell growth *in vitro* and *in vivo* (McKie et al., 2012; Wu and Sood, 2012). Recently, the advances in structure-function relationships of OPCML give rise to its potential as an anti-cancer therapy (Birtley et al., 2019). DNA methylation is the most common epigenetic modification and is widely reported for the transcriptional silencing of tumor suppressor genes in ovarian cancer. Previous studies have indicated that OPCML methylation is associated with the inactivation of OPCML, which has a tumor suppressor function in ovarian cancer (Simovic et al., 2019). However, studies with small sample sizes and different control types and methylation methods might produce inconsistent results. For example, in the study performed by Czekierdowski et al. (2006) OPCML methylation was observed in 20 out of 43 cases of ovarian cancer, but no methylation was found in four normal ovaries. No significant association was observed in this study. The control sample size in this study was small. Additionally, most previous studies reported the methylation frequency of OPCML in tumor and non-tumor tissues or serum, but the precise estimates for the associations were unclear. In the present study, we attempted to consolidate the available data and found that OPCML methylation was associated with an increased risk of ovarian cancer. The findings from the sensitivity analysis showed that our results are reliable and stable. Overall, given the TSG role and frequent methylation inactivation of OPCML in ovarian cancer, the restoration of OPCML expression could be a promising approach for ovarian cancer treatment.

In the subgroup analysis, the OR was 38.17 in China but 7.85 in Poland. The cause may be due to the limited studies in Poland. Therefore, more studies should be performed to confirm these observations in Europeans. In the serum subgroup, the OR value was highest (OR = 102.86) than that in the tissue subgroup (OR = 18.22). The results should be interpreted with caution because of the relatively small number of subjects included in the serum subgroup. Given that serum is a promising biomarker for non-invasive ovarian cancer diagnosis, further well-designed studies are warranted to explore the diagnostic performance of OPCML methylation in serum. Regarding the methods, the OR was 21.78 in the MSP group and 139.45 in the restriction enzyme-related analysis group. Technically, MSP amplify either methylated (M) or unmethylated (U) alleles after bisulfite conversion and evaluate methylation status in CpG regions (Herman et al., 1996). MSRE-PCR can recognize and degrade unmethylated DNA sequences whereas the methylated DNA sequences remain intact (Ramsahoye et al., 1997). Taken together, the results of subgroup analyses showed that OPCML methylation was significantly associated with an increased risk of ovarian cancer, regardless of the sample size, sample type, control type, and methods. Additionally, we found that the methylation status was associated with III/IV stage and poorly differentiated tumors. This finding suggests that OPCML is related to the malignant progression of ovarian cancer.

Based on publicly available datasets, we observed that the DNA methylation levels of cg16639665, cg23236270, and cg15964611 were consistently higher in cancer tissues than that in normal tissues, which validated some results of our meta-analysis. However, we did not confirm the difference of the OPCML methylation levels in different groups of clinical stage, grade, and so on. We found the positive association between DNA methylation level of cg25853078 in OPCML promoter region and OPCML expression. This positive association did not fit the hypothesis that DNA methylation levels in promoter region suppress the expression and contribute to ovarian cancer development. The DNA methylation levels in previous published papers were regions but not one CpG site. There are some CpG sites in OPCML promoter region, but we only assessed one CpG site in the promoter region based on the HumanMethylation27 platform. Therefore, other CpG sites were not calculated based on the TCGA data, which may have significant roles in the regulation of OPCML expression. Our study possessed several limitations. First, the total sample size in our study was relatively small and enrolled studies had low quality. Second, all the eligible studies were case-control designs and we could not explore the causation between OPCML methylation and ovarian cancer risk. Additionally, we used the incidence of OPCML methylation in the case and control groups to evaluate the pooled estimates because the adjusted ORs were not reported in the original studies. Further prospective studies with large samples are warranted to determine the role of OPCML in ovarian cancer risk and progression.

## Data Availability Statement

The original contributions presented in the study are included in the article/Supplementary Material, further inquiries can be directed to the corresponding authors.

## Author Contributions

YS, KX, and JW: conception and design. YS and JK: acquisition of data. HX, XW, YC, WL, JH, and DL: analysis and interpretation of data. YS, KX, and JW: manuscript drafting or revising. All authors contributed to the article and approved the submitted version.

## Conflict of Interest

The authors declare that the research was conducted in the absence of any commercial or financial relationships that could be construed as a potential conflict of interest.
