# Supplementary material for: OPCML Methylation and the Risk of Ovarian Cancer: A Meta and Bioinformatics Analysis
Source: Front Cell Dev Biol. 2021 Mar 11;9:570898. doi: 10.3389/fcell.2021.570898 (PMC7990783; doi:10.3389/fcell.2021.570898)
Supplement: Supplementary file 1 [file Table_1.DOC]

| Supplementary Table 1. Quality of included 7 papers. | | | | | | | | | | | | | |
| --- | --- | --- | --- | --- | --- | --- | --- | --- | --- | --- | --- | --- | --- |
| Articles | Year |  | Selection | |  |  | Comparability | |  | Exposure | | | NOS |
| (authors, year) |  | 1 | 2 | 3 | 4 |  | a | b |  | 1 | 2 | 3 | score |
| Wang et al [19] | 2015 | * | * | - | * |  | - | - |  | * | * | * | 6 |
| Xing et al [20] | 2015 | * | * | - | * |  | - | - |  | * | * | * | 6 |
| Zhou et al [25] | 2014 | * | * | - | * |  | - | - |  | * | * | * | 6 |
| Zhou et al [22] | 2014 | * | * | - | * |  | - | - |  | * | * | * | 6 |
| Czekierdowski et al[24] | 2006 | * | * | - | * |  | - | - |  | * | * | * | 6 |
| Zhang et al [21] | 2006 | * | * | - | * |  | - | - |  | * | * | * | 6 |
| Liu et al [26] | 2008 | * | * | - | * |  | - | - |  | * | * | * | 6 |
| ‘*’ indicates NOS quality assessment star awarded. ‘ -’ indicates that no star was awarded. | | | | | | | | | | | | | |
